# Velopharyngeal Insufficiency and Impaired Tongue Movement Indicate Neuromuscular Disorders: A 10-Year Statistical Study in a Single Tertiary Institution

**DOI:** 10.3390/jcm14020477

**Published:** 2025-01-13

**Authors:** Aiko Fujisaki, Rumi Ueha, Carmel Cotaoco, Misaki Koyama, Taku Sato, Takao Goto, Kenji Kondo, Tatsuya Yamasoba

**Affiliations:** 1 Department of Otolaryngology, and Head and Neck Surgery, Faculty of Medicine, The University of Tokyo, Tokyo 113-8655, Japan; aiko223915@gmail.com (A.F.); mel_cotaoco@yahoo.com (C.C.); m.sekiguchi.tymc9@gmail.com (M.K.); taku.koro.z@gmail.com (T.S.); gottytakao@gmail.com (T.G.); kondok-tky@umin.ac.jp (K.K.); tyamasoba-tky@umin.ac.jp (T.Y.); 2 Department of Otolaryngology, Tokyo Teishin Hospital, Tokyo 102-8798, Japan; 3 Swallowing Center, The University of Tokyo Hospital, Tokyo 113-8655, Japan; 4 Ear Nose Throat Head and Neck Surgery Institute, The Medical City, Metro Manila 1600, Philippines

**Keywords:** neuromuscular disorders, velopharyngeal closure insufficiency, dysphagia, voice disorders, dysarthria

## Abstract

**Objectives**: This study aimed to determine the positive predictive value of our NMD Suspicion Criteria in the diagnosis of NMDs. Other clinical factors routinely examined in our voice and swallowing examinations were also investigated to see if they had a significant association with the diagnosis of NMDs. **Methods**: This study retrospectively investigated the medical charts of patients who visited our Voice and Swallowing outpatient clinic between 2013 and 2022. Patients with previously diagnosed NMDs were excluded from the analysis. Among the remaining patients, we included those that were referred to neurologists for further evaluation due to suspicion of having an NMD based on the NMD Suspicion Criteria. The patients were then divided into groups according to the status of their diagnosis within 2 years of referral as “diagnosed”, “denied”, or “observed”. These three groups of patients were then compared according to the following clinical findings; velopharyngeal insufficiency (VPI), tongue atrophy, impaired tongue movement, dysarthria, vocal fold mobility impairment, dysphagia, involuntary movement, gait disturbances, weight loss, and a sense of fatigue in order to see if they were significantly associated with the diagnosis of NMDs. **Results**: Of 3769 outpatients without a confirmed diagnosis of NMDs, 37 were referred to neurologists for suspected NMDs, and 19 (51%) were diagnosed with NMDs. VPI and impaired tongue movement were significant diagnostic factors for NMDs (*p* = 0.014, 0.033). VPI during speech (*p* = 0.045) was more strongly associated with the diagnosis of NMDs than VPI during swallowing (*p* = 0.076). Fatigue was a significant related factor for other diseases (non-NMDs) causing Voice and Swallowing problems (*p* = 0.049). **Conclusions**: In the outpatient clinic setting, suspicion of NMD should be raised, particularly when VPI and impaired tongue movement are observed, prompting a thorough assessment of velopharyngeal closure during both speech and swallowing.

## 1. Introduction

Patients with a wide range of symptoms are referred to otolaryngology outpatient clinics by family physicians. Some of these patients that present with dysphagia, dyspnea, hoarseness, or other vocalization difficulties, in rare instances, have undiagnosed neuromuscular disorders (NMDs) underlying their symptoms; however, there are few data on the percentage of patients with undiagnosed NMDs accessing general outpatient services. The decision of when to refer patients to specialists, such as neurologists, based on clinical observations is undeniably at the discretion of the examining physician.

NMDs that may initially present with dysphagia include Parkinson’s disease (PD), amyotrophic lateral sclerosis (ALS), myotonic dystrophy (MD), and others [1,2,3]. On the other hand, disorders that initially present with hoarseness or other vocalization difficulties and articulation problems include PD, ALS, multiple system atrophy (MSA), and myasthenia gravis (MG) [4,5,6,7,8]. PD is one of the most prevalent diseases in the world, and it often leads to dysarthria and impaired tongue movement [9]. These impairments significantly impact communication and swallowing in patients with PD, characterized by decreased coordination and speed in chewing, substantially prolonged oropharyngeal transit time, and delayed initiation of swallowing [2,10]. In ALS, which is, in general, less prevalent than PD, damage to the hypoglossal, vagus, and glossopharyngeal nerves—whether nuclear or supranuclear—can cause muscle and nerve atrophy. These lead to tongue and velopharyngeal atrophy, impaired pharyngeal contraction, and subsequent dysarthria and dysphagia [8,11,12].

Velopharyngeal insufficiency (VPI) is a condition in which the movement and coordination of the soft palate and pharyngeal wall are impaired, leading to incomplete closure during speech and swallowing [13]. VPI can occur in ALS, multiple sclerosis, Guillain–Barré syndrome, MG, MD, etc. [14,15]. Dysarthria is a motor speech disorder caused by damage to the neural pathways that control the facial, oral, and pharyngolaryngeal muscles. Smooth speech relies on the coordination of the motor system, including the basal ganglia and cerebellar circuits. When even a single component of this system is impaired, it results in difficulties with articulation, phonation, and resonance [16]. Impaired tongue movement is a common symptom in various neurological disorders, affecting speech, swallowing, and overall quality of life. The main neurological conditions associated with impaired tongue movement include ALS, PD, MG, and Guillain–Barré syndrome [17,18]. Vocal fold mobility impairment is more likely to be identified in NMDs such as PD, MSA, and ALS [19]. Patients with NMDs often exhibit weight loss and fatigue, which are associated with swallowing difficulties and muscle weakness [20,21]. Fatigue is a common and debilitating symptom across a wide range of NMDs, with a complex underlying pathophysiology.

Physicians in our Voice and Swallowing clinic often receive referrals to evaluate the swallowing function of patients with diagnosed NMDs and have therefore gained substantial clinical experience in this area. Using this considerable experience and combining it with the data found in the literature regarding the common symptoms seen in patients with NMDs, we have formulated the NMD Suspicion Criteria. These criteria include unique clinical findings that we have observed in patients with diagnosed NMDs. Patients that consult at our Voice and Swallowing clinic due to dysphagia and/or voice and articulation problems who meet these criteria are then referred to a neurologist so they may be further evaluated for the presence of an NMD.

In this study, we retrospectively investigated patients from our Voice and Swallowing clinic who were suspected of having an NMD based on our criteria and were then referred to a neurologist for further evaluation. This study aimed to assess the validity of these criteria by determining their positive predictive value in diagnosing NMDs and identifying the clinical findings that contribute to the diagnosis of NMDs.

## 2. Materials and Methods

### 2.1. NMD Suspicion Criteria

In our institution, we evaluate the following items as part of the NMD suspicion criteria: the presence of VPI, dysarthria, tongue atrophy, impaired tongue movement, vocal fold mobility impairment, dysphagia, involuntary movement, gait disturbance, weight loss, and fatigue. Patients were considered as having weight loss if they lost 10% or more of their body weight in one half year and as having fatigue if they had general malaise as a subjective symptom. After excluding malignancy or stroke as the cause, patients who satisfied two or more criteria, including at least one major criterion, were referred to a neurologist, as shown in Table 1.

### 2.2. Patients and Ethics

We included patients who visited the Voice and Swallowing outpatient clinic of the University of Tokyo Hospital between 2013 and 2022 with complaints of dysphagia, dyspnea, voice disorders, or dysarthria. The study protocol was approved by the Human Ethics Committee of the University of Tokyo (no. 2487, 2022179NI, approval date 31 October 2022), and complied with the tenets of the amended Declaration of Helsinki. Written informed consent or an opt-out form on the website was obtained from every patient, and patient anonymity was preserved.

### 2.3. Methodology

We conducted a retrospective single-center study using the medical charts from our hospital database. We reviewed the clinical and demographic profiles of the subjects, including age, sex, presence of an NMD diagnosis, and referral to a neurologist.

First, we investigated the positive predictive value of being diagnosed with an NMD after meeting two or more criteria of the NMD suspicion criteria, including at least one major criterion. We reviewed the charts of all the patients who presented to the Voice and Swallowing clinic due to dysphagia, dyspnea, voice disorders, or dysarthria. All patients who had already been diagnosed with an NMD were excluded from the analysis. Among the remaining patients, we extracted those that were referred to the Department of Neurology after having met two or more criteria of the NMD suspicion criteria, including at least one major criterion, and for whom a diagnosis of malignancy or stroke were excluded. Next, we checked the status of these patients’ diagnoses within 2 years of referral to confirm if they had been diagnosed with an NMD. They were classified as either diagnosed, denied, or observed. Those classified as “diagnosed” were those who were eventually confirmed to have an NMD, “denied” were those who were eventually confirmed to not have an NMD, and “observed” were those still undergoing observation with no current definitive diagnosis.

Second, other clinical factors that are routinely assessed during our swallowing evaluation were examined. These are findings that are not part of the current NMD suspicion criteria and include laryngeal sensation, laryngeal elevation, pharyngeal contraction, and swallowing initiation. The charts of patients who had been referred to neurologists were investigated to check the findings of the previously stated clinical factors. The findings of these factors were then compared between the diagnosed, denied, and observed groups and statistically examined to see if there was an association with the diagnosis of an NMD.

Endoscopic laryngeal examinations were used to evaluate VPI, vocal fold mobility, and laryngeal sensation. According to the method previously reported [22], VPI was evaluated during both speech and swallowing. VPI was considered present if velopharyngeal closure was incomplete during either speech or swallowing, whereas VPI was considered absent if velopharyngeal closure was complete during either speech or swallowing. To assess VPI during speech, patients were instructed to say their name, date of birth, and a sentence with frequent “ka” column sounds. For swallowing, VPI was evaluated by having the patient perform two dry swallows. Vocal fold mobility and laryngeal sensation were evaluated via a graded assessment, employing a three-point scale (0—no impairment; 1—mild impairment; 2—severe impairment) to quantify the extent of impairment (Table 2). Pharyngeal contraction, laryngeal elevation, and swallowing initiation were assessed using videofluoroscopic swallowing studies (VFSSs). Based on the video recordings, two or more otolaryngologists with over 10 years of experience in dysphagia management rated the degree of these impairments on a three-point scale with a score of 0–2 (Table 2).

### 2.4. Statistical Analyses

We analyzed all data using BellCurve for Excel (version 4.03; Social Survey Research Information Co., Ltd., Tokyo, Japan) and examined the associations among clinical and demographic profiles for the diagnosis of NMDs. We used the Kruskal–Wallis test for multiple comparisons and the post hoc Scheffe test to analyze continuous variables, and the Chi-Square test and further residual analyses to analyze categorical variables. Statistical significance was set at *p* < 0.05.

## 3. Results

**Figure 1:**
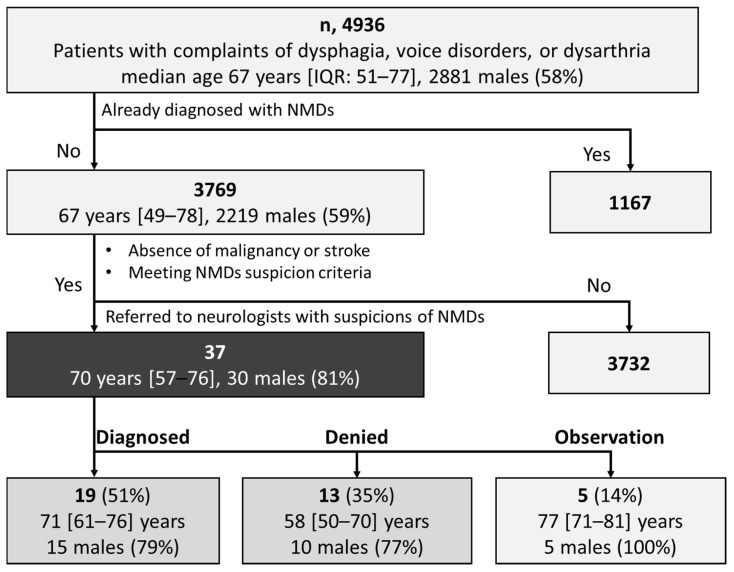
Flowchart of patient selection. n: number; IQR: interquartile range; NMDs: neuromuscular disorders.

Figure 1 shows the demographic data of all 4936 patients (2881 males, 58%; median age, 67 years; interquartile range [IQR] (51–77) years) who were eligible and included in this study. Of these, 1167 patients (662 males, 57%; 67 [56–76] years) had already been diagnosed with NMDs, while 3769 patients (2219 males, 59%; 67 [49–78] years) had not received a diagnosis of an NMD. Among the undiagnosed patients, 37 (30 males, 81%; 70 [57–76] years) were referred to a neurologist with suspicion of NMDs during outpatient examinations, comprising 0.98% of the undiagnosed patients. Within this group, 19 patients (51%) received a confirmed diagnosis of NMDs (diagnosed group), 13 patients (35%) received a negative diagnosis (denied group), and 5 patients (14%) were under ongoing observation without a definitive diagnosis (observed group) (Figure 1). This demonstrated that the positive predictive value of the NMD suspicion criteria is higher than 50%.

To summarize, of all patients who presented to the Voice and Swallowing outpatient clinic with complaints of dysphagia, dyspnea, voice disorders, or dysarthria, 0.4% were discovered to have undiagnosed NMDs. Among patients who had not yet been diagnosed with NMDs at the time of their visit to the Voice and Swallowing outpatient clinic, 0.5% were later diagnosed with NMDs.

The most prevalent primary disease was ALS, which was diagnosed in seven patients, followed by PD and MG, which were each diagnosed in two patients. Other diagnoses included multiple system atrophy, muscular dystrophy, essential tremor, and facial onset sensory and motor neuronopathy (Table 3).

The association between each clinical finding and the diagnosis of NMDs was examined among the diagnosed, observed, and denied groups. VPI (*p* = 0.014) was significantly associated with NMDs. VPI during speech (*p* = 0.045) was more strongly associated with the diagnosis of NMDs than VPI during swallowing (*p* = 0.076). Impaired tongue movement was also a significant factor associated with NMDs (*p* = 0.033). Tongue atrophy, involuntary movements, dysarthria, and weight loss were more frequently observed in the diagnosed group than in the denied group. In contrast, a perceived sense of fatigue was significantly more common in the denied group than in the diagnosed group (*p* = 0.049). The impairments of pharyngolaryngeal findings listed in Table 2 were scored and compared among the diagnosed, denied, and observed groups; however, none of them were found to be significant clinical factors contributing to the diagnosis of NMDs (Table 4).

Regarding VPI, which is a significant prognostic factor for NMDs, 11 of the 37 patients with suspected NMDs were noted to have VPI. Of these 11, 9 patients were diagnosed with NMDs, and 2 patients were strongly suspected to have an NMD but were under observation to form a definitive diagnosis. ALS was the most prevalent disease diagnosed (five patients). Intriguingly, VPI was not necessarily observed concurrently during both phonation and swallowing. Patients with VPI discrepancies between speech and swallowing included four patients with VPI only during speech (two ALS and two others) and three patients with VPI only during swallowing (one ALS, one MG, and one other) (Table 5).

## 4. Discussion

The present study demonstrated that 0.50% of patients (19/3769 patients) who presented to the Voice and Swallowing outpatient clinic with dysphagia, dyspnea, voice disorders, and dysarthria as the chief complaint had undiagnosed NMDs as an underlying disease. Additionally, using our original NMD suspicion criteria, over 50% of patients suspected to have an NMD were indeed diagnosed within two years with an NMD after evaluation by a neurologist. The significant clinical findings that predicted NMDs were VPI and impaired tongue movement. Notably, VPI can occur either during speech or swallowing and not necessarily during both speech and swallowing.

In patients with NMDs, it often takes time from the onset of the initial symptoms to the ultimate confirmation of diagnosis, such as 16–18 months for ALS [23], 3–10 years for PD [24], and 3.8 years for MSA [25]. With the development of medications, such as riluzole for ALS [26] and levodopa and dopamine agonists for PD [27], early diagnosis could play a crucial role in improving the quality of life of these patients. In the case of MG, treatment methods such as plasma exchange therapy and intravenous immunoglobulin infusion therapy [28] could lead to early improvement in patients’ symptoms if the diagnosis is made early. Therefore, patients should be examined by a specialist as soon as possible in order to expedite the diagnostic process from the onset of symptoms and allowing them to receive earlier treatment. Unfortunately, there are few data on the percentage of patients with undiagnosed NMDs consulting general outpatient clinics. The decision of when to refer patients to specialists, such as neurologists, based on clinical observations is undeniably at the discretion of the examining physician. Considering this study revealed that VPI and impaired tongue movement were significant predictors of NMDs, family physicians and general otolaryngologists should consider the possibility of underlying NMDs when evaluating patients with speech and/or swallowing disorders, especially if they present with the aforementioned clinical findings. Otolaryngologists in particular should assess velopharyngeal function during both speech and swallowing when conducting endoscopic evaluations of the pharyngolaryngeal area and swallowing function. If VPI is observed, an underlying NMD should be taken into account. Along with fiberoptic examinations, approaches such as videofluoroscopy, tongue and pharyngeal pressure measurements, and nasalance measurements can be applied to assess pharyngeal function in cases of suspected NMDs [29].

In this study, we discovered that 0.5% of patients who presented with speech and swallowing difficulties at our Voice and Swallowing clinic were later found to have underlying NMDs. Given that the majority of patients referred to neurologists based on the NMD suspicion criteria were diagnosed with NMDs within two years, these criteria show potential as a supportive tool in clinical practice. As no other similar criteria have been reported, the effectiveness of this study’s NMD suspicion criteria warrants further examination.

### 4.1. Velopharyngeal Insufficiency in Neuromuscular Disorders

The nasal and oral cavities must be completely closed during speech, whistling, blowing, swallowing, and vomiting [18,30]. Velopharyngeal closure is accomplished through the contraction of several velopharyngeal muscles, including the levator veli palatini, tensor veli palatini, musculus uvulae, superior pharyngeal constrictor, palatopharyngeus, and palatoglossus [18]. Motor innervation for velopharyngeal closure is supplied primarily through the trigeminal nerve (cranial nerve V), facial nerve (VII), and pharyngeal plexus of nerves (IX and X) [31]; however, it has also been suggested that not all cranial nerves supplying the pharyngeal plexus are involved in motor innervations of the velopharyngeal muscles [32]. Knowing this, the condition of VPI may vary depending on the site and degree of damage to the nerves and muscles associated with velopharyngeal closure. It is not surprising to encounter individual variations in impairments, especially in patients with NMDs. VPI can occur in multiple sclerosis, Guillain–Barré syndrome, MG, MD, etc. [14]. In this study, patients with VPI were later diagnosed with ALS, MG, or MD.

VPI is a significant symptom experienced by many patients with ALS, particularly those with bulbar involvement. VPI affects both speech and swallowing functions in ALS. It was previously reported that 26–46% of ALS patients with bulbar symptoms exhibit VPI [22,33]. VPI in patients with ALS can result in a range of complications affecting both speech and swallowing. Speech-related issues may include a nasal quality to the voice, impaired articulation, and difficulties in controlling tone [34,35]. Swallowing difficulties associated with VPI include the regurgitation of food and liquids into the nasal cavity, reduced pharyngeal contractility, and challenges in effectively managing food and saliva, all of which can significantly impact the patient’s quality of life [36]. Fiberoptic laryngoscopy is recognized as a critical diagnostic tool for investigating VPI in ALS. It offers the ability to detect distinctive features such as speech–swallow dissociation in velopharyngeal function and asymmetrical weakness in the pharyngeal constrictor muscles, which are typically not evident through general neurological evaluation [22].

VPI in MG results from the autoimmune nature of the disease, which impairs the function of muscles responsible for velopharyngeal closure. The autoimmune attack at the neuromuscular junction disrupts nerve–muscle communication, leading to muscle weakness, particularly in the soft palate and pharyngeal muscles. Fatigable muscle weakness, a hallmark of MG, exacerbates VPI symptoms with prolonged use, often worsening throughout the day. The fluctuating nature of MG further contributes to variable VPI severity [37,38,39]. There are limited data on the prevalence of VPI in MG. VPI was detected in 50% of MG patients with bulbar symptoms, but not at all in those without bulbar symptoms [37], and was found in around 1% of children diagnosed with MG [39]. This points to a higher prevalence of VPI among those with bulbar involvement in MG. It is important to note that VPI in MG is a functional issue due to muscle weakness rather than a structural problem. The severity can vary among patients and may improve with appropriate MG treatment [37].

VPI in MD is mainly caused by the neuromuscular dysfunction of the disease, which leads to muscle weakness and dysfunction, particularly affecting the muscles responsible for velopharyngeal closure. VPI can occur at various stages of MD, and it may even be the presenting symptom in some cases [15,40,41,42]. While previous reports indicate that VPI is a recognized and potentially common feature in MD, the exact frequency has not been reported. Further research would be needed to determine the precise prevalence of VPI in patients with MD.

As a point of interest, it is quite possible that the findings of velopharyngeal closure may differ during speech and swallowing. Previous studies have indicated that ALS should be suspected when there is a discrepancy between velopharyngeal closure during swallowing and speech [22,43]. However, findings from this study indicate that this is not a unique finding in ALS but is also present in other NMDs. Thus, VPI discrepancy between swallowing and speech does not seem specific to ALS.

### 4.2. Tongue Mobility Impairment in Neuromuscular Disorders

Tongue mobility impairment is a common issue in various neuromuscular disorders, affecting speech, swallowing, and oral function. It can result from muscle weakness (as seen in ALS, MG, and MD), neurological impairments (e.g., PD and ALS), or structural changes (such as tongue enlargement in Duchenne muscular dystrophy) [17,44,45]. Key manifestations include dysarthria, dysphagia, fatigability, and atrophy. Specific disorders present with distinct tongue dysfunctions, such as slowed tongue movements in PD, fatigable weakness in MG, fasciculations in ALS, and enlarged tongues in MD. Diagnostic tools such as videofluoroscopy, ultrasound, and tongue pressure measurements are used for diagnosis. Early recognition and assessment are crucial for preventing complications like aspiration and malnutrition [45,46]. In ALS, a decline in tongue motor function results in reduced range and speed of tongue movements [47]. Similarly, in muscular dystrophy, a decrease in oral motor function, including that of the tongue, leads to impairments in mastication and swallowing [48].

### 4.3. Other Clinical Findings in Neuromuscular Disorders

In this study, there was no significant association between tongue atrophy or involuntary movements and the diagnosis of NMDs; however, the small number of cases may have influenced these results.

Tongue atrophy is a significant clinical finding in various NMDs, each presenting with distinct features and implications. In MG, tongue atrophy occurs in approximately 5–15% of patients and is characterized by a rare triple-furrowed tongue [46,49,50]. This condition has the potential for reversibility with treatment, even after extended periods, and can occur in different MG subtypes, including acetylcholine receptor antibody-positive, muscle-specific receptor tyrosine kinase antibody-positive, and seronegative forms [49]. In contrast, ALS leads to the rapid degeneration of tongue function, with significant weakness and reduced thickness. Tongue atrophy is observed in the initial phases of ALS [51], and the progression of tongue dysfunction in ALS is faster compared to other NMDs [44]. Late-onset Pompe Disease also involves tongue atrophy, which is associated with weakness and distinct sonographic abnormalities, such as fibrofatty replacement, setting it apart from other acquired or hereditary myopathies [52]. While less common, tongue atrophy has been reported in Lambert–Eaton myasthenic syndrome, often in a reversible form, although this was previously unreported in the condition [53].

Involuntary movements, including resting tremors, are common symptoms in NMDs and vary depending on the condition. Resting tremors occur when muscles are at rest and persist even when supported, typically slowing or disappearing with voluntary movement. They are most commonly linked to Parkinson’s disease, Parkinsonism, and certain medications, such as antipsychotics and anti-nausea drugs [51,54]. These tremors usually affect the hands but can also involve the legs, chin, lips, or tongue, often presenting as a “pill-rolling” motion. Tremors can worsen with stress, fatigue, or certain medications [55,56]. Other types of involuntary movements in NMDs include essential tremor, dystonia, chorea, ataxia, and myoclonus [57]. Essential tremor is primarily an action or postural tremor, usually affecting the hands, head, or voice [58]. Dystonia is characterized by sustained muscle contractions that lead to twisting or repetitive movements [57,58]. Chorea involves brief, irregular, dance-like movements that flow from one body part to another [59]. Ataxia affects coordinated movement, causing clumsiness and balance problems. Myoclonus is marked by sudden, brief, shock-like involuntary movements. Each of these movements presents distinct challenges and symptoms depending on the underlying disorder.

Patients with NMDs often exhibit weight loss owing to reduced food intake associated with swallowing difficulties and muscle weakness [20,21]. However, considering that weight loss is also evident in diseases such as malignant tumors, endocrine metabolic disorders, sarcopenia, and frailty [60], it is understandable that weight loss was not a significant factor associated with NMDs in this study. Fatigue is a common and often debilitating symptom in various NMDs, including ALS, MD, Guillain–Barré syndrome, and Pompe disease. It can be categorized into experienced fatigue and physiological fatigue, and may have both central and peripheral origins [61,62,63]. While fatigue was not identified as a contributing factor to the diagnosis of NMDs in this study, it is postulated that weight loss and fatigue resulting from conditions other than NMDs may have impacted the outcomes.

### 4.4. Limitations and Future Research Directions

This study has several limitations. This retrospective chart review was limited by incomplete or missing documentation. A multivariate analysis could not be performed to verify the results because of the small sample size. The evaluation of VPI was limited to endoscopic findings, and incorporating nasalance measurements might have uncovered more instances of VPI [64]. Patients who were not diagnosed with NMDs at the time of this study but may develop the disease in the future were not included in the statistical analysis. The potential for developing NMDs later on cannot be ruled out among patients who were not suspected of having an NMD using the NMD suspicion criteria at the time of consultation.

A retrospective study of the 3732 patients who were not referred to neurologists might have helped determine the negative predictive value by investigating whether any of them were later diagnosed with NMDs. However, the large number of subjects and the high likelihood that many no longer followed up at our institution made it difficult to track them. A prospective multi-center study should be planned in the future to evaluate the validity and effectiveness of the NMD suspicion criteria.

A multidisciplinary approach is essential for the early diagnosis and subsequent treatment of patients with NMDs [65]. Given the critical role of otolaryngologists in evaluating VPI, we strongly recommend close collaboration between neurologists and otolaryngologists in both the clinical management and future research of NMDs.

## 5. Conclusions

In cases where patients present to otolaryngologists with complaints of swallowing difficulties or speech impairments, consideration must be given to the possibility of an underlying neuromuscular disorder, even though they are not very common. In particular, when VPI and tongue mobility impairment are observed, neuromuscular disorders should be suspected, prompting a thorough assessment of velopharyngeal closure during both speech and swallowing.

## Figures and Tables

**Table 1:** NMD suspicion criteria; NMD: neuromuscular disorder.

NMD Suspicion Criteria	
Major criteria	Velopharyngeal insufficiency
	Tongue atrophy
	Impaired tongue movement
	Dysarthria
Minor criteria	Dysphagia
	Vocal fold mobility impairment
	Involuntary movement
	Gait disturbances
	Weight loss
	Fatigue
Refer patients to a neurologist when (1) they meet at least two criteria, including at least one major criterion, and (2) when a malignant tumor or stroke has been ruled out as the etiology of symptoms.	

**Table 2:** Grading scale of pharyngolaryngeal findings.

	Score 0	Score 1	Score 2
	Normal	Mildly Impaired	Severely Impaired
Vocal fold mobility	Well	Limited vocal fold movement	Completely immobile vocal fold
Laryngeal sensation	Well	Less responsive to contact stimulation	Unresponsive to contact stimulation
Laryngeal elevation	Well	Impaired laryngeal elevation on swallowing	Little or no laryngeal elevation on swallowing
Pharyngeal contraction	Well	Mildly impaired contraction	Little or no contraction
Swallowing initiation	Intact	Delayed swallowing initiation	Absent or severely delayed swallowing initiation

**Table 3:** Diagnosis of neuromuscular disorders. no.: number.

Diagnosis of Neuromuscular Disorders	19 Patients
Amyotrophic lateral sclerosis, no (%)	7 (37%)
Parkinson’s disease, no. (%)	2 (11%)
Myasthenia gravis, no. (%)	2 (11%)
Other, no. (%)	8 (42%)

**Table 4:** Association between clinical findings and diagnosis of neuromuscular disorders. no.: number; IQR: interquartile range. *: *p* < 0.05.

	Diagnosed	Observation	Non-Diagnosed	*p* Value
Patients, no. (%)	19 (51%)	5 (14%)	13 (35%)	
Age, years, median [IQR]	71 [61–76]	77 [71–81]	58 [50–70]	0.069
Male, no. (%)	15 (79%)	5 (100%)	10 (77%)	0.50
Endoscopic findings, no. (%)				
Velopharyngeal insufficiency (VPI)	9 (47%)	2 (40%)	0 (0%)	0.014 *
VPI during speech	7 (36%)	1 (20%)	0 (0%)	0.045 *
VPI on swallowing	5 (26%)	2 (40%)	0 (0%)	0.076
Vocal fold mobility impairment (score)	0 [0–0]	0 [0–0]	0 [0–0]	0.3
Impaired laryngeal sensation (score)	0 [0–0]	0 [0–0]	0 [0–1]	0.34
Videofluoroscopic swallow study score, median [IQR]				
Reduced pharyngeal contraction	1 [1–1]	1 [1–1]	1 [0–1]	0.50
Impaired laryngeal elevation	0 [0–1]	1 [1–1]	0 [0–1]	0.26
Delayed swallowing initiation	1 [0–1]	0 [0–1]	0 [0–1]	0.82
Oral findings, no. (%)				
Tongue atrophy	5 (26%)	0 (0%)	0 (0%)	0.065
Impaired tongue movement	6 (32%)	0 (0%)	0 (0%)	0.033 *
Other findings, no. (%)				
Involuntary movement	10 (53%)	1 (20%)	2 (15%)	0.071
Gait disturbances	4 (21%)	2 (40%)	1 (8%)	0.28
Dysarthria	10 (53%)	4 (80%)	3 (23%)	0.067
Weight loss	6 (32%)	3 (60%)	1 (8%)	0.067
Fatigue	0 (0%)	0 (0%)	3 (23%)	0.049 *

**Table 5:** Details of patients with velopharyngeal insufficiency. no.: number.

Velopharyngeal Insufficiency	
Patients, no.	11
Only during swallowing, no. (%)	4 (36%)
Only during speech, no. (%)	3 (27%)
Both, no. (%)	4 (36%)
Diagnosis of neuromuscular disorders (NMDs)	
Amyotrophic lateral sclerosis, no. (%)	5 (45%)
Myasthenia gravis, no. (%)	1 (9%)
Myotonic dystrophy, no. (%)	1 (9%)
Facial onset sensory and motor neuronopathy, no. (%)	1 (9%)
Frontotemporal lobar degeneration, no. (%)	1 (9%)
No confirmed diagnosis, but strongly suspected NMDs, no. (%)	2 (18%)
Velopharyngeal insufficiency only during swallowing, no.	4
Amyotrophic lateral sclerosis, no. (%)	2 (50%)
Facial onset sensory and motor neuronopathy, no. (%)	1 (25%)
Frontotemporal lobar degeneration, no. (%)	1 (25%)
Velopharyngeal insufficiency only during speech, no.	3
Amyotrophic lateral sclerosis, no. (%)	1 (33%)
Myasthenia gravis, no. (%)	1 (33%)
No confirmed diagnosis, but strongly suspected NMDs, no. (%)	1 (33%)

## Data Availability

The datasets used and analyzed during the current study are available from the corresponding author on reasonable request.
